# A Scalable Framework for Longitudinal Surveillance of Research Evolution in Digital Medicine

**DOI:** 10.21203/rs.3.rs-11210969/v1

**Published:** 2026-10-20

**Authors:** Yilun Liang, Gongbo Zhang, Casey Ta, Yilu Fang, Chunhua Weng

## Abstract

We developed a novel scalable framework that tracks the evolution of research topics across many journals and applied it to biomedical informatics, a field central to digital medicine, contributing methods for semantic harmonization across heterogeneous publication metadata. We analyzed 78,073 articles from 29 PubMed-indexed journals (2011–2025) carrying 95,872 distinct keywords: 75% author-supplied via PubMed or publishers, 24% substituted MeSH descriptors, 1% generated from abstracts. Three overlapping waves emerged: genomics and foundational informatics, machine learning, and generative AI. Attention shifted in 34 of 70 methodology and 22 of 86 health topics; rises persisted on author-supplied keywords alone, whereas most declines disappeared, implying that changes in keyword source, not waning attention, explain most apparent declines. Digital health technologies was the connective topic, clinical and computational collaboration grew, and the Journal of Biomedical Informatics bridged the journal communities. The framework can be applied to other biomedical research fields to identify emerging research priorities.

